# 

**Published:** 1915-05-01

**Authors:** 


### Buildings Contemplated.

Aberavon.—The foundation-stone of a new hospital has been formally laid. The estimated cost is £3,333.

Dundee.—The Parish Council have purchased the large mansion known as Ashcliffe, to be used as a children's hospital.

Grassington.—The Health Committee recommend the Bradford Corporation to accept tenders amounting to £23,701 for building a sanatorium at Grassington.

Jarrow.—The Town Council have adopted a scheme for extending the hospital, which involves the purchase of five acres of land at a cost of £200 an acre, and a total estimated expenditure of £14,000.

Gloucester.—The Gloucestershire County Council have authorised the Committee of Visitors to prepare a scheme for a new epileptic block for sixty patients and the necessary staff. The estimated cost is £10,000.

### The Book Market.

LIST OF NEW BOOKS RECEIVED FROM THE FOLLOWING PUBLISHERS:—

Smith, Elder and Co.: "The Minor Horrors of War." By A. E. Shipley, Sc.D. 1s. 6d. net.

### Pamphlets and Papers.

"Year-Book of the City of Buenos Aires, 1913."

The Truth about the Sino-Japanese "Conversations." The Chinese Students' Union, 36 Bernard Street, W.C.

Massachusetts General Hospital: "Home-made Hospital Furniture." By Dr. Frederic A. Washburn.

"Six Years' Experiences at the Medico-mechanical Department of the Massachusetts General Hospital." By C. Hermann Bucholz, M.D., Chicago.

"Hospital Records." By Joseph B. Howland, Assistant Administrator, Massachusetts General Hospital. "Relation of the Staff to the Administration of Hospitals." By David L. Edsall, M.D., Boston. Reprinted from "The Modern Hospital."

"Morale Fondée sur les Lois de la Nature." By M. Deshumbert. Watts and Co., 17 Johnson's Court, Fleet Street, E.C.

### Personalia.

Mr. W. Long has been appointed Chairman of the Establishment Sub-Committee of the London Insurance Committee.

Miss M. K. Gibson, M.B., B.Ch., B.A.O. (R.U.I.), has been appointed by the Metropolitan Asylums Board an assistant medical officer in the Hospitals Service.

### Admiralty Doctors.

CONSULTANTS AND RETAINING FEES.

Commander Bellairs put the following Parliamentary question to the First Lord in regard to consultants employed by the Admiralty:

Whether any sums of over £3,000 a year are being paid to doctors and surgeons in civil life as retainers for their services; if so, in what cases and what amounts; and whether the arrangement was made prior to the war and allows of private practice as well?

Dr. Macnamara replied: The following consultants are paid over £3,000 per annum: Mr. G. L. Cheatle, C.B., C.V.O., F.R.C.S., £5,000 per annum; Sir W. W. Cheyne, Bt., C.B., F.R.C.S., £5,000 per annum; Mr. Raymond Johnson, M.B., F.R.C.S., £5,000 per annum; Sir W. Macewen, F.R.C.S., £5,000 per annum; Mr. H. D. Rolleston, M.D., F.R.C.S., £5,000 per annum; Mr. G. R. Turner, F.R.C.S., £5,000 per annum.

The employment of eight consultants was approved before the outbreak of war. The whole time of these consultants is at the disposal of the Admiralty, and while private practice is not forbidden, it must not in any way interfere with the performance of an officer's naval duties.

**Subscriptions**, which may commence at any time, are payable in advance. Cheques and Post-Office Orders should be crossed London County and Westminster Bank, Covent Garden Branch, and made payable to the Manager of The Hospital.

Rates of Subscription (payable in advance).

| | | |
|---|---|---|
| UNITED KINGDOM. | | |
| Three Months | (including Postage) ... ... ... ... | 2s. 0d. |
| Six Months | do. ... ... ... ... | 4s. 0d. |
| Twelve Months | do. ... ... ... ... | 6s. 6d. |
| FOREIGN AND COLONIAL. | | |
| Three Months | (including Postage) ... ... ... ... | 2s. 6d. |
| Six Months | do. ... ... ... ... | 5s. 0d. |
| Twelve Months | do. ... ... ... ... | 8s. 8d. |

**Rates of Subscription:** Twelve months, 6/6; Six months, 4/-; Three months, 2/-, post free to any address in the United Kingdom. Abroad, Twelve months, 8/8; Six months, 5/-; Three months, 2/6, post free.—Address The Subscription Dept., "The Hospital," The Hospital Building, 28/2 Southampton Street, Strand, London, W.C.

